# War Cases of Osteitis

**Published:** 1919

**Authors:** 


***War Cases of Osteitis***. By MM. DUMAS and MALARTIC, *La Presse Médicale,* October 3, 1918.

The authors have dealt with more than 600 cases of fistulae subsequent to war-wounds.

The treatment given consisted in opening widely the focus of osteitis, and cleansing according to the classic method.

As post-operatory treatment, all antiseptics generally in use were employed, together with muscular and cartilaginous grafting.

The authors practised some partial primary sutures, and, in a few cases, total secondary sutures. They resorted to cutaneous grafts, heliotherapy, hot air and local baths of oxygen, to help in the process of cicatrization.

The best results were obtained in leaving the wound wide open, letting the cicatrization progress spontaneously, and using Dakin-Carrel solution as disinfecting agent.

628 cases were treated in this way. One proved fatal and a few will never recover. All the others have left the hospital with closed fistulae and good scars.

